# Economic evaluations of community health worker programs focussed on neglected tropical diseases in low- and middle-income countries (2015–2024): A scoping literature review

**DOI:** 10.1371/journal.pgph.0005551

**Published:** 2025-12-05

**Authors:** Linnea Stansert Katzen, Madelyn Miyares, Kelsey Vaughan, Cleo Baskin, Madeleine Ballard, Maryse Kok, Ariwame Jimenez, Matias Iberico, Jessica Cook, Angele Bienvenue Ishimwe, Lily Martin, Patrick Kawooya, Zeus Aranda, Molly Mantus, Meghan Bruce Kumar, Karen E. Finnegan, Sandra Mudhune, Mardieh Dennis, Daniel Palazuelos, Dickson Mbewe, Michee Nshimayesu, Paul Revill, James O’Donovan

**Affiliations:** 1 Community Health Impact Coalition, London, United Kingdom; 2 Institute for Life Course Health Research, Department of Global Health, Faculty of Medicine and Health Sciences, Stellenbosch University, Cape Town, South Africa; 3 Centre for Health and Sustainability, Department of Women’s and Children’s Health, Uppsala University, Uppsala, Sweden; 4 Department of Health Policy and Management, Gillings School of Global Public Health, University of North Carolina at Chapel Hill, North Carolina, United States of America; 5 Bang for Buck Consulting, Amsterdam, Netherlands; 6 ResiliSUS lab, Fundação Oswaldo Cruz, Rio de Janeiro, Brazil; 7 Arnhold Institute for Global Health, Icahn School of Medicine at Mount Sinai, New York, New York, United States of America; 8 Department of Clinical Sciences, Liverpool School of Tropical Medicine, Liverpool, United Kingdom; 9 Malawi-Liverpool Wellcome Trust Clinical Research Programme, Blantyre, Malawi; 10 Compañeros En Salud, Ángel Albino Corzo, México; 11 Tulane University School of Medicine, New Orleans, United States of America; 12 Partners in Health, Boston, United States of America; 13 TIP Global Health, Kigali, Rwanda; 14 NYU Health Sciences Library, NYU Langone Health, New York, United States of America; 15 Nama Community Wellness Center, Mukono, Uganda; 16 Last Mile Health, Boston, United States of America; 17 Department of Nursing, Midwifery and Health, Northumbria University, Newcastle upon Tyne, United Kingdom; 18 KEMRI-Wellcome Trust Programme, Nairobi, Kenya; 19 Department of Global Health and Social Medicine, Harvard Medical School, Boston, United States of America; 20 Pivot, Ranomafana, Madagascar; 21 Lwala Community Health, Nairobi, Kenya; 22 Last Mile Health, Monrovia, Liberia; 23 Division of Global Health Equity, Brigham and Women’s Hospital, Harvard Medical School, Boston, United States of America; 24 Kasungu District Hospital, Kasungu, Malawi; 25 University of Global Health Equity, Kigali, Rwanda; 26 Centre for Health Economics, University of York, York, United Kingdom; PLOS: Public Library of Science, UNITED STATES OF AMERICA

## Abstract

Neglected tropical diseases (NTDs) are a diverse group of more than twenty diseases caused by parasitic, bacterial, and viral infections, affecting more than one billion individuals worldwide. Economic evidence can help guide the investment in Community Health Workers (CHWs) who can help expand access to preventive and curative NTD services in low- and middle-income countries (LMICs). A scoping review was conducted across ten databases and grey literature, covering studies published between August 2015 and July 2024. Search terms related to “Community Health Workers” and “Economic Evaluations” were used. Studies were screened via Covidence software based on inclusion and exclusion criteria. Data on study methodology, costs, and outcomes were extracted, tabulated in Microsoft Excel, and analysed. Of the 29 included scenarios (n = 10 studies), 7 were about community mass drug administration and 22 focused on other topics - such as disease-specific prevention and treatment (e.g., dengue). Across scenarios, the most commonly reported outcomes were cost per service delivered (ranging from $0.13-$5.33) and cost per capita (ranging from $10.24-$21.09). Five scenarios reported on cost-effectiveness, with varied results (40–50% of scenarios were reported as cost effective). One study found that interventions were more likely to be cost-effective when they leveraged integrated care as opposed to vertical approaches. The evidence base for economic evaluations regarding CHW involvement in NTD programs is highly limited. From the 10 studies identified there was no clear conclusion with regards to cost-effectiveness or affordability of CHWs in NTD programs in LMICs. To better understand the critical role CHWs can play in both prevention- and treatment-focused NTD programs, further evidence of the cost-effectiveness and affordability of such interventions is needed.

## Introduction

Neglected tropical diseases (NTDs) are a diverse group of more than twenty conditions, including those caused by parasitic, bacterial, viral, and fungal infections, as well as specific non-communicable diseases, affecting more than one billion individuals worldwide, with over 70% of the burden of disease being in low- and middle-income countries (LMICs) [1,2]. These diseases are endemic in tropical and subtropical regions [2], and most prevalent in rural areas affected by poverty, inadequate sanitation, limited healthcare access, and weak health systems [3]. NTDs both stem from and perpetuate poverty, as affected families often face catastrophic health spending and financial hardship due to high out-of-pocket expenses when seeking care, combined with reduced ability to participate in economic activities due to ill-health [4]_._

It is estimated that NTDs result in the loss of around 17 million Disability-Adjusted Life Years (DALYs) each year [5]. Though NTDs account for the second-highest burden of DALYs among infectious diseases - behind only HIV/AIDS - they have historically received less global attention and funding than other major infectious diseases [1,2]. For example, in a recent analysis of 29 LMICs, total NTD spending by government and partners averaged 1.3% of infectious disease control expenditure, compared to 14% for HIV [2,6].

To address this, several key initiatives were launched. The 2012 World Health Organization (WHO) NTD Roadmap and the 2012 London Declaration both helped to bring global attention and funding to NTDs, highlighting their debilitating effects on individuals and communities, including physical disability, societal stigma, and economic hardship. [7,8] The 2012 Roadmap established measurable goals for the control, elimination, and eradication of NTDs by 2020, and these were later expanded in the 2021–2030 Roadmap with a broader scope, new targets, and strategic shifts [2].

While support for NTD prevention and treatment has grown - for example, $777 million was raised at the 2023 Reaching the Last Mile Forum to be invested in combatting certain NTDs [9] - most funding goes toward Mass Drug Administration (MDA) for prevention than to case management (CM-NTDs) for treatment, despite an identified need for both approaches [2]. This is because CM-NTDs require more time and resources, including diagnosis and follow-up care. Consequently, many countries continue to implement disease-specific, vertical programs centered on MDA for prevention, often neglecting the needs of those already affected by NTDs. The updated WHO Roadmap for 2021–2030 seeks to address this gap by emphasizing the integration of NTD services to enhance access to case management and improve treatment outcomes. Since 2016, the Sustainable Development Goals (SDGs) include a target related to ending NTDs alongside AIDS, tuberculosis and malaria by 2030 [1,10].

The 2025 global health funding cuts are expected to significantly hinder progress toward achieving these targets and disproportionately affect community health worker (CHW) programs, which are vital in the fight against NTDs [11]. CHWs play a crucial role in delivering essential healthcare services, particularly in remote and underserved areas where access to medical facilities is limited [11]. For NTDs, this involves delivering prevention and treatment services in remote areas, including MDA programs, active case-finding, health education, and referrals to primary and secondary health facilities. This is further supported by the WHO Roadmap for 2021–2030, which specifically calls for the involvement of CHWs in NTD control [2]. While definitions of CHWs vary by country and program [12], they are generally recognized as lay or semi-professional health workers who receive targeted training to provide preventive, promotive, and basic curative services within their communities [13]. CHWs bridge the gap between communities and formal healthcare systems, offering health education, disease surveillance, treatment adherence support, and in some cases, direct administration of medicines [14].

Yet, despite the important role for CHWs, there remains a paucity of evidence evaluating the costs and affordability of CHW involvement in NTDs. This evidence is particularly important for government decision makers (including stakeholders in Ministries of Health and Finance). The most recent scoping review about the costs and cost-effectiveness of CHWs in LMICs by Vaughan et al. (2015) did not include any studies about NTDs, nor did it capture findings about the affordability of CHWs [15].

This current scoping review aims to address this gap in the literature by providing an updated overview of the evidence on the costs, cost-effectiveness, and affordability of CHW programs for NTDs in LMICs between 2015–2024. Additionally, it assesses the methodologies used in these evaluations and examines how costs, cost-effectiveness, and affordability are reported. This research intends to contribute to a better understanding of the role CHWs can play in helping countries achieve the targets set out in the WHO Roadmap to prevent, control and eliminate or eradicate NTDs [2].

## Methods

An initial wider scoping review was conducted across ten databases and grey literature, covering studies published between August 2015 and July 2024, to identify and map the available evidence on economic evaluations of both vertical and integrated horizontal CHW programmes in LMICs. Search terms related to “Community Health Workers” and “Economic Evaluations” were used (full search terms available in S1 Table). The PRISMA ScR can be found in the S1 Checklist.

Studies were screened via Covidence software based on predefined inclusion and exclusion criteria. Eligible studies were those that primarily evaluated CHW programmes (excluding those related to other health professionals), focused on vertical CHW programmes addressing NTDs, and reported an economic evaluation, either full or partial. Interventions had to be located in LMICs according to World Bank classifications for the costing year. Studies were excluded if they were commentaries, protocols, editorials, conference abstracts, or systematic reviews (though reference lists were searched). Evaluations of digital add-ons to CHW programmes were also excluded. No restrictions were applied by study quality, language, or timeframe of analysis. The full PICO framework can be found in the S2 Table.

In keeping with the scoping review approach, no formal quality appraisal was conducted. Studies were not excluded on methodological grounds, reflecting the intent to capture the range of available evidence. Data on study methodology, costs, and outcomes were extracted independently by two reviewers, tabulated in Microsoft Excel, and standardized to 2024 US dollars where possible. Outcomes and cost measures were categorized into predefined domains to facilitate comparison. Data were synthesised narratively and descriptively due to the heterogeneity of interventions, settings, and evaluation methods. For full methods, see our published paper in this article series [16,17].

Due to the large number of studies identified and the heterogeneity between studies, reporting of results has been divided into several publications, by disease area or type of CHW, for clarity and to facilitate comparisons between similar studies. This paper focuses exclusively on vertical CHW programs focused on NTDs, including Buruli ulcer; Chagas disease; dengue and chikungunya; dracunculiasis; echinococcosis; foodborne trematodiases; human African trypanosomiasis; leishmaniasis; leprosy; lymphatic filariasis; mycetoma, chromoblastomycosis and other deep mycoses; noma; onchocerciasis; rabies; scabies and other ectoparasitoses; schistosomiasis; soil-transmitted helminthiases; snakebite envenoming; taeniasis/cysticercosis; trachoma; and yaws [18].

### Patient and public involvement

Patients and the public were not consulted as part of this scoping review.

### Ethics approval

A self-assessment was conducted via the University of Washington Human Subjects Institutional Review Board (IRB) which determined that this study was not human subjects research and did not require IRB review. A reflexivity statement can be found in the S2 Checklist.

## Results

### Search results

**Fig 1:**
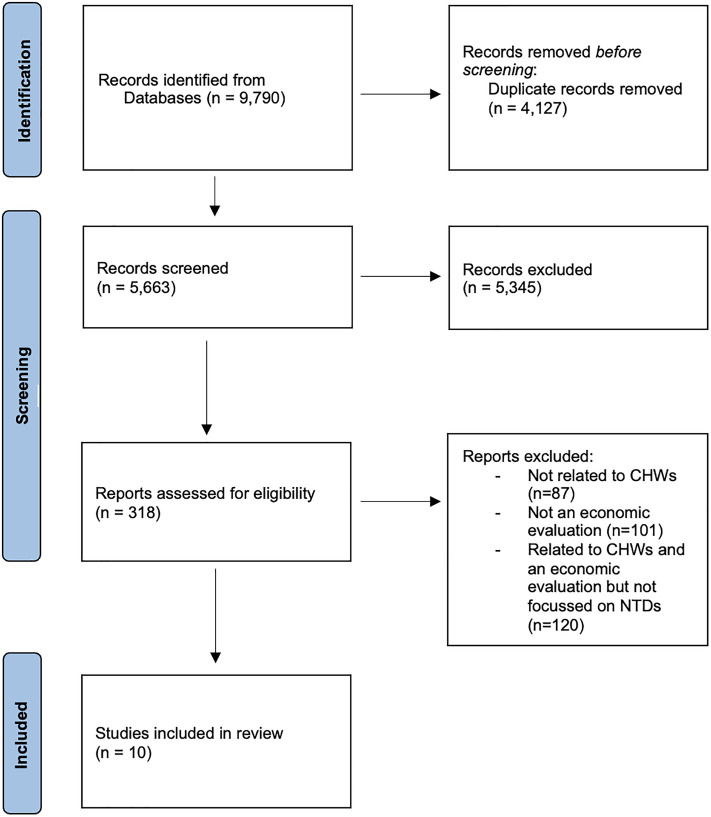
PRISMA flow diagram. A total of 9,790 records were identified through database searches. After removing 4,127 duplicates, 5,663 records were screened. Of 318 full-text reports assessed for eligibility, 10 studies met the inclusion criteria. Exclusions were based on relevance to CHWs, presence of economic evaluation, and NTD focus.

The initial broader literature search (which included NTDs, but also other health areas such as non-communicable diseases (NCDs), mental health, HIV, Malaria, TB [16], and reproductive, maternal, newborn and child health (RMNCH) as well as horizontal, integrated CHW programs [17], (HIV, TB, Malaria, horizontal recently published, while others are currently all under peer-review) yielded 9,790 articles, reduced to 5,663 after the removal of duplicates. 5,345 studies were excluded following abstract screening, and an additional 170 were excluded after full-text review. After coding studies by disease area, this process resulted in 10 NTD studies being included in this review. The studies covered 29 individual scenarios. These scenarios are further detailed in Table 1. Further details can be found in the PRISMA flow chart (Fig 1) [29].

**Table 1:** Details of CHW roles and scenarios in NTD management.

Population and type of CHW	Intervention type	NTDs studied	Nature of intervention (Prevention, curative, both)	Scenarios descriptions	Role of CHW	Comparator
Factors influencing mass drug administration adherence and community drug distributor opportunity costs in Liberia: a mixed-methods approach [19]						
Rural, NTD-endemic communities in Liberia Salaried CHWs (Community Drug Distributors) delivering MDA for NTDs	cMDA	Unspecified	Both	One scenario (n = 1)	Salaried CHWs conducting MDA for lymphatic filariasis, schistosomiasis, and onchocerciasis	N/A
A mixed methods approach to evaluating community drug distributor performance in the control of neglected tropical diseases [20]						
Rural communities in Uganda with high burden of lymphatic filariasis, schistosomiasis, trachoma, and onchocerciasis Volunteer CDDs implementing an integrated NTD program	cMDA	Lymphatic filariasis, schistosomiasis, trachoma, onchocerciasis	Both	One scenario (n = 1)	Volunteer CHWs performing integrated preventive chemotherapy including drug distribution, register maintenance, monitoring, and reporting Annual campaign	N/A
Costs of community-wide mass drug administration and school-based deworming for soil-transmitted helminths: evidence from a randomised controlled trial in Benin, India and Malawi [21]						
Mixed rural and peri-urban populations in Benin, India, and Malawi at risk of soil-transmitted helminths Mass drug distribution of deworming drugs	cMDA	Soil-Transmitted Helminthiases	Prevention	Three scenarios (n = 3) reporting findings from Benin, India and Malawi	CHWs distributing albendazole via door-to-door and school-based delivery for soil-transmitted helminths	Community wide vs. school-based distribution
Mass drug administration campaigns: comparing two approaches for schistosomiasis and soil-transmitted helminths prevention and control in selected Southern Malawi districts. [22]						
Residents of rural districts in Southern Malawi Mass drug distribution to prevent schistosomiasis	cMDA	Soil-Transmitted Helminthiases and Schistosomiasis	Prevention	Two scenarios (n = 2) reporting on two approaches for prevention and control. Community directed distribution vs. standard care partnering with Health Surveillance Assistants	CHWs distributing drugs for schistosomiasis and soil-transmitted helminths prevention	Standard intervention for prevention (with Health Surveillance Assistants) schistosomiasis vs. Community Directed Distribution
Economic analysis of dengue prevention and case management in the Maldives [23]						
Residents of inhabited and resort islands in the Maldives at risk of dengue virus infection Home visits to screen for and treat dengue fever	Other (home visiting for Dengue)	Dengue fever	Both	One scenario (n = 1)	CHWs conducting home visits for dengue screening and treatment	n/a
Cost-effectiveness and social outcomes of a community-based treatment for podoconiosis lymphoedema in the East Gojjam zone, Ethiopia [24]						
Adults affected by or at risk of podoconiosis lymphoedema in rural East Gojjam Zone, Ethiopia Community-based treatment for podoconiosis lymphoedema	Other (treatment through monthly meetings)	Podoconiosis lymphoedema	Curative	One scenario (n = 1)	CHWs holding monthly meetings on podoconiosis lymphoedema prevention and care	CHW-based intervention vs. no intervention
Community-directed interventions are practical and effective in low-resource communities: experience of ivermectin treatment for onchocerciasis control in Cameroon and Uganda, 2004–2010 [25]						
Remote, low-resource communities in Cameroon and Uganda endemic for onchocerciasis CHW-led distribution of ivermectin for treatment of onchocerciasis	Other (distribution of ivermectin)	Onchocerciasis	Both	Four scenarios (n = 4) reporting on years 2007 and 2010 in Cameroon and Uganda, respectively	CHWs using community-directed ivermectin distribution for onchocerciasis	Cameroon vs Uganda
Financial and economic costs of the elimination and eradication of onchocerciasis (River Blindness) in Africa [26]						
Populations in 28 sub-Saharan African countries with varying levels of onchocerciasis endemicity Treating onchocerciasis with ivermectin	Other (distribution of ivermectin)	Onchocerciasis	Both	Twelve scenarios (n = 12) reporting on 2013 and 2045 costs, on financial and economic costs, and control, elimination, and eradication respectively	CHWs distributing ivermectin, mobilizing communities, and conducting censuses for onchocerciasis	Control vs. elimination vs. eradication
Cost-effectiveness of community mobilization (Camino Verde) for dengue prevention in Nicaragua and Mexico: A cluster randomized controlled trial [27]						
Urban and semi-urban households in dengue-endemic regions of Nicaragua and Mexico Community mobilization for dengue prevention	Other (community intervention for Dengue)	Dengue fever	Prevention	Two scenarios (n = 2) reporting on findings from Mexico and Nicaragua	CHWs conducting community mobilization for dengue prevention	Community intervention vs. standard care (government-initiated vector control methods)
Assessing the cost‐effectiveness of integrated case management of Neglected Tropical Diseases in Liberia [28]						
Communities in Liberia affected by Buruli ulcer, leprosy, lymphatic filariasis morbidity, and yaws Integrated case management of NTDs	Other (integrated case management)	Buruli ulcer, leprosy, lymphatic filariasis morbidities, and yaws	Curative	Two scenarios (n = 2) reporting on CHWs and other healthcare workers screening for NTDs during two MDA periods yearly, and b) CHWs doing door-to-door visits daily to screen and provide education	CHWs conducting door-to-door NTD screening and education during MDA periods	Standard care (fragmented screening and treatment of NTDs, also by CHWs) vs integrated management with consistent door to door visits for screening and information

### Results reporting

Each section describes the CHW programs and alternatives assessed and reports on the relevant cost, cost-effectiveness and affordability findings as reported by authors, with cost findings converted to 2024 US$ to facilitate comparisons [30,31]. For cost-effectiveness findings, we report whether authors compared CHW models against an alternative service or delivery modality, such as facility-based care, as well as any benchmark used to determine cost-effectiveness (e.g., whether a cost-effectiveness threshold based upon willingness to pay or GDP per capita was used). For affordability, we note whether authors reported how the intervention affects the overall healthcare budget (budget impact analysis), including whether the intervention is affordable within the current budget constraints. That said, we report on cost-effectiveness and affordability based on the authors’ determination or conclusions from the original study, regardless of whether a threshold was used. Findings are reported by scenario; each scenario represents a unique combination of CHW characteristics (paid/unpaid), setting and methodological characteristics. For instance, a paper reporting two costs per service, one representing volunteer CHWs and one CHW labour at a specified cost, is presented as two scenarios. Likewise, a paper reporting cost-effectiveness of CHW programs separately for three different countries is presented as three scenarios.

### Study characteristics

We identified ten studies focused on NTD-related topics and approaches for prevention and treatment, across five low-income countries (Ethiopia, Liberia, Malawi, Nicaragua, and Uganda) [19–25,27,28], three lower-middle income countries (Benin, Cameroon and India) [21,25], and two-upper-middle income countries (Maldives and Mexico) [23,27]. One study spanned across 27 African countries (Table 1) [26]. The targeted NTDs included lymphatic filariasis, schistosomiasis, trachoma, onchocerciasis, soil-transmitted helminthiases, dengue fever, podoconiosis-related lymphoedema, Buruli ulcer, leprosy, and yaws.

The size of the interventions in the included studies was only reported in six scenarios and varied from 15 to 556 CHWs [20,22,24,25]_._ The number of beneficiaries ranged from 659 to 6,285,000 persons [20,22–24,26,27]_._ In five scenarios CHWs were salaried [19,21,24,28] with two studies reporting the specific salary amount ($56 and $90 per month) [19,24]_._ In 14 scenarios (from two studies) CHWs were volunteers, [22,26] in two scenarios they received a stipend, [20,27] and in one they were valued at the level of other health care workers [27]_._ For the remaining scenarios (n = 7), CHW remuneration was not specified (see Table 2 for full details) [21,23,25]_._ Three of the scenarios used standard care as a comparator [22,27,28], seven compared different approaches against each other [21,24]_,_ four compared findings between countries [25], and twelve compared various scenarios of onchocerciasis being controlled, eliminated, and eradicated against one another [26]. Three scenarios had no comparator [19,20,23].

**Table 2:** Summary details of 10 included studies on CHW NTD programs.

Country	Type of Economic Analysis	Population served	CHWs (#)	Compensation method (2024 US$)	Cost/ service (2024 US$)	Additional cost outcomes (2024 US$)	Cost-effectiveness conclusion* (threshold used)	Affordability conclusion (criteria used)
Factors influencing mass drug administration adherence and community drug distributor opportunity costs in Liberia: a mixed-methods approach [19]								
Liberia	Partial - Cost analysis	Not reported	Not reported	Salaried ($56/month)	n/a	n/a	Not assessed	Not assessed
A mixed methods approach to evaluating community drug distributor performance in the control of neglected tropical diseases [20]								
Uganda	Partial - Cost analysis	34,615	64	$1.80/day stipend (only paid during training days)	n/a	n/a	Not assessed	Not assessed
Costs of community-wide mass drug administration and school-based deworming for soil-transmitted helminths: evidence from a randomised controlled trial in Benin, India and Malawi [21]								
Benin	Partial - Cost analysis	Not reported	Not reported	Not reported	$2.66	Cost per unit of cMDA ($2.56)	Not assessed	Not assessed
India	Partial - Cost analysis	Not reported	Not reported	Not reported	$1.31	Cost per unit of cMDA ($1.44)	Not assessed	Not assessed
Malawi	Partial - Cost analysis	Not reported	Not reported	Salaried (not documented)	$5.38	Cost per unit of cMDA ($6.20)	Not assessed	Not assessed
Mass drug administration campaigns: comparing two approaches for schistosomiasis and soil-transmitted helminths prevention and control in selected Southern Malawi districts [22]								
Malawi	Partial - Cost analysis	25,893 - 28,764	25 and 140 in the two approaches respectively	No (volunteers)	n/a	n/a	Not assessed	Not assessed
Economic analysis of dengue prevention and case management in the Maldives [23]								
Maldives	Partial CEA - cost description	1.8 million	Not reported	Not reported	Not reported	Cost/ capita per year ($10.56)	Not assessed	Not assessed
Cost-effectiveness and social outcomes of a community-based treatment for podoconiosis lymphoedema in the East Gojjam zone, Ethiopia [24]								
Ethiopia	Full CEA	659	15	Salaried ($90.47/month)	Not reported	n/a	Mixed, dependent on criteria used (comparison with alternative)	Not assessed
Community-directed interventions are practical and effective in low-resource communities: experience of ivermectin treatment for onchocerciasis control in Cameroon and Uganda, 2004–2010 [25]								
Cameroon	Partial -Cost description	Not documented	212	Not reported	Not reported	Cost/ CHW ($1.46-$3.55)	Not assessed	Not assessed
Uganda	Partial -Cost description	Not documented	556	Not reported	Not reported	Cost/ CHW ($0.72-$4.2)	Not assessed	Not assessed
Financial and economic costs of the elimination and eradication of onchocerciasis (River Blindness) in Africa [26]								
Angola, Benin, Burkina Faso, Burundi, Cameroon, Central African Rep., Chad, Congo, Rép. Côte d’ivoire, Equatorial Guinea, Ethiopia, Gabron, Ghana, Guinea, Guinea-Bissau, Liberia, Malawi, Mali, Mozambique, Nigeria, Senegal, Sierra Leone, South Sudan, Sudan, Tanzania, Togo, Uganda	Partial CEA - cost description	6,285,000	Not documented	No (volunteers)	$0.13 - $5.33	n/a	Not assessed	Not assessed
Cost-effectiveness of community mobilization (Camino Verde) for dengue prevention in Nicaragua and Mexico: A cluster randomized controlled trial [27]								
Nicaragua	Full - CEA	19,326	Not documented	Other (valued at same level as other cadre of health professionals, $13.66/month)	n/a	Cost/capita per year ($10.24)	No (GDP per capita)	Mixed
Mexico	Full CEA	15,839	Not documented	Stipend (not documented)	n/a	Cost/capita per year ($21.09)	Yes - marginally cost-effective (GDP per capita)	Mixed
Assessing the cost‐effectiveness of integrated case management of Neglected Tropical Diseases in Liberia [28]								
Liberia - integrated care intervention	Full CEA	Not documented	Not documented	Salaried (not documented)	n/a	Cost per patient diagnosed ($714) Cost per patient on treatment ($ 1,151)	Yes (comparison with alternative)	Not assessed
Liberia - vertical intervention (control)	Full CEA	Not documented	Not documented	Salaried (not documented)	n/a	Cost per patient diagnosed ($ 3,942) Cost per patient on treatment ($ 13,800)	No (comparison with alternative)	Not assessed

* As reported by the authors. Commonly used thresholds such as GDP per capita have faced criticism for failing to consider local resource availability, such as health opportunity costs, and for being less useful in decision-making since it often results in most interventions being labelled as cost-effective.

N/A: not applicable.

### Intervention types

Four studies, representing 13 scenarios, evaluated community-wide mass drug administration (cMDA) [19–22], which can be both a preventive and/or curative intervention. These included drug delivery through door-to-door campaigns, school-based deworming, and community-directed distribution. Six studies, representing 16 scenarios, focused on non-cMDA CHW approaches to NTD management, which may be prevention- or treatment-focused [23–28]. These included home visits, prevention through educational community meetings, and integrated case management. Details of CHW intervention scenarios are presented in Table 1.

### Roles of CHWs

CHWs were involved in various roles in the NTD management: screening for the NTDs (n = 23 scenarios) [19–21,25,26,28], delivery of medication (n = 23 scenarios) [19–22,25,26] administrative tasks (n = 18 scenarios) [19,20,25,26], outreach and training (n = 9 scenarios) [19,22–24,27,28] and ongoing management (n = 3 scenarios) [19,23,28].

### Cost outcomes

Five scenarios represented full economic evaluations, [24,27,28] while 24 utilized partial economic evaluations [19–23,25,26]. The most common cost outcomes were cost per service (n = 15 scenarios), ranging from $0.13-$5.38 [21,26] and cost per capita (n = 3 scenarios) [20,27] ranging from $10.24-$21.09. Other cost outcomes included cost per CHW (n = 2 scenarios) [25]_,_ ranging from $0.72-$4.20, cost per cMDA dose (n = 3 scenarios) [21], ranging from $1.44-$6.20, cost per patient diagnosed (n = 2 scenarios) [28], ranging from $714-$3,942, and cost per patient on treatment (n = 2 scenarios) [28], ranging from $1,151-$13,800. These last two cost outcomes came from the same study, and represent CHWs performing integrated case management of NTDs in Liberia reducing diagnostic costs per patient fivefold and treatment costs tenfold versus vertical programs [28].

### Cost-effectiveness findings

Of five scenarios reporting on cost-effectiveness, 40% were cost-effective, rising to 50% after excluding scenarios with unclear findings [24,27,28]. One concluded that CHWs were cost-effective compared to no intervention when considering the number of acute dermatolymphangioadenitis (ADLA) episodes and the dermatology quality of life index (DLQI), but less costly and less effective when using WHO Disability Assessment Schedule (WHODAS) 2.0 criteria. [24,32]. The study which compared community mobilization for dengue fever prevention in Mexico and Nicaragua found the Mexican scenario to be cost-effective, while the Nicaragua scenario was not, according to the authors due to Nicaragua’s low per capita GDP and the high cost of grant-funded management personnel [27]. The study comparing NTD management approaches in Liberia concluded that integrated care with daily door-to-door visits to screen and inform about NTDs was more cost-effective than the alternative (screening only during two time periods annually) [28].

### Affordability findings

Only one study representing two scenarios reported on affordability [27]. It found that the community mobilization for dengue prevention was not affordable as implemented, but suggested that with administrative efficiencies, a multivalent approach, and incorporating the benefits of reducing other arboviral diseases, it could become more affordable, though no specific affordability criteria were used [27]. The overall paucity of affordability data highlights a significant gap in the evidence base for assessing the economic feasibility of such interventions.

### Economic evaluation methods features

In this section we summarise selected methods-related findings across all included studies (n = 10) and scenarios (n = 29).

Across both studies focusing on cMDA and on non-cMDA CHW approaches to NTD management, the most commonly reported outcomes were cost per service (n = 15) and cost per capita (n = 3). Four scenarios (n = 4) reported cost per CHW, and three (n = 3) reported cost per cMDA dose. We did not find any studies which reported on cost per DALY averted.

Of the 29 scenarios, only ten (n = 10) reported on the perspective used: four took a health system perspective, three took a provider perspective, and three took a mixed perspective. Seventeen scenarios employed a time horizon greater than one year, while eleven scenarios employed a one-year time horizon. The majority of scenarios reported on resources used for the intervention and included training costs (n = 25), non-training capital items (meaning items used over one year, such as equipment) (n = 21), recurrent costs (n = 23) and indirect costs or overheads (n = 19).

Only two studies (n = 5 scenarios) reported on cost-effectiveness, comparing CHWs against no intervention (n = 1) and against GDP per capita thresholds (n = 2), with mixed conclusions. Only one study (n = 2 scenarios) reported on affordability, with mixed conclusions and no clear criteria.

None of the studies used the Consolidated Health Economic Evaluation Reporting Standards (CHEERS) checklist, despite it being the leading guidance for authors to follow to ensure that health economic evaluations are identifiable, interpretable, and useful for decision making.

## Discussion

In this current review we have summarized evidence from 10 studies (involving 29 scenarios) published between 2015 and 2024 on economic evaluations of CHWs’ involvement in NTD management. Compared to the 2015 review, [15] the number of studies focused on economic evaluation of CHW involvement in NTD control has increased, given that no economic evaluations on NTDs were reported previously. Of the five scenarios reporting on cost effectiveness, 40% were found cost-effective, rising to 50% when excluding scenarios with unclear conclusions.

A critical finding of this review is the limited reporting on cost-effectiveness and affordability of CHW-led interventions for NTDs, despite the significant burden that NTDs place on health systems in LMICs. Only two studies (five scenarios) reported on cost-effectiveness, and just one study (two scenarios) addressed affordability. Notably, none of the cMDA-focused studies reported on either of these measures. This evidentiary gap is significant given that cost-effectiveness and affordability are foundational to informing health policy and investment decisions, particularly in resource-constrained settings where CHW programs to control NTDs are often deployed. MDA (not specifically delivered by CHWs) has already been proven cost-effective in other settings for certain NTDs, such as for soil-transmitted helminths [33]. Further evidence on CHWs roles in these initiatives can guide policymakers in designing supportive frameworks for CHW engagement in NTD control.

The few studies that explored cost-effectiveness yielded mixed conclusions that were sensitive to context and methodology. For example, CHWs delivering monthly education sessions for podoconiosis lymphoedema appeared cost-effective when measured using disease-specific outcomes, but results varied depending on the outcome metric (DLQI and WHODAS 2.0 criteria) [32]. Similarly, community mobilisation for dengue prevention was found to be marginally cost-effective in Mexico but not in Nicaragua. Plausible explanations for this are - differences in economic context, differences in implementation costs and effectiveness and differences in funding mechanisms and requirements. The use of GDP per capita thresholds in these evaluations has well-documented limitations, including failing to consider local resource availability, poor reflection of health opportunity costs and limited usefulness for decision-makers seeking to allocate finite budgets [34,35]_._ Importantly, no study used cost per DALY averted - a widely accepted metric that enables comparison across diseases and interventions. The absence of this standardised outcome measure limits the ability to benchmark the value of CHW-delivered NTD interventions against alternative health investments and hinders their inclusion in national or global priority-setting exercises.

Regarding the affordability of CHW interventions for NTDs, only one study (two scenarios) reported on affordability, with mixed conclusions. Authors suggested that the previously-mentioned dengue prevention interventions in Mexico and Nicaragua were unlikely to be affordable currently, but could become so in the future - though without specifying against which criteria this assessment was made. Future studies should incorporate clear, standardized criteria such as budget impact analyses, including both short- and long-term financial implications for health systems and communities, considering varying implementation scenarios. Wider economic evidence on approaches to control NTDs, for example mass distribution of medication and vector management (not necessarily involving CHWs), has found that interventions to end NTDs “are affordable” for the governments of most endemic countries, given that treatment and vector control combined require less than 0.1 percent of domestic health spending, and assuming that medicines for some NTDs are donated by pharmaceutical companies [36]_._ Despite this low share, some countries have not allocated any part of the health expenditure to NTDs in the past [6] leading WHO to repeat calls for increased expenditure not just by governments, but by donors and pharmaceutical companies as well [2]. Given the recent significant cuts in international aid and donor funding, there are serious risks to progress in NTD control. The current financial climate also highlights the need for alternative financing mechanisms, such as increased domestic financing.

The lack of robust cost-effectiveness and affordability analyses of CHW-led NTD interventions may reflect the lack of historical investment in the prevention and treatment of NTDs in general, leading to fewer studies assessing the economic viability of CHW-led interventions. This has direct implications for policy and practice. Without such evidence, decision-makers are left without critical information needed to assess whether these interventions provide good value for money or are financially feasible at scale. This could delay or derail the uptake of potentially impactful strategies.

Another important finding, and caveat, from this review is that in 14 of the 29 scenarios analyzed, CHWs were volunteers, which complicates efforts to accurately assess the cost-effectiveness and affordability of interventions as a key element of costs (i.e., salaries) and effectiveness (i.e., productivity) is missing [37]. This limitation is significant, as volunteer-based CHW programs often rely on unpaid labor, potentially leading to exploitation and undervaluation of their contributions [38,39]_._ Best practice guidelines on CHW programming emphasize the importance of fair compensation for CHWs [40]. If evaluations continue to assume volunteer CHWs, they may underestimate the true cost of sustainable programs, potentially misleading decision-makers. Where CHWs are not compensated in line with guidelines, researchers should explore analyses which value them appropriately.

One additional important point to note is that this scoping review focuses on vertical approaches to managing NTDs. While vertical programs offer targeted efficiency, they often operate in isolation from broader health systems. In contrast, integrated approaches, which combine interventions for multiple disease areas or embed them within general healthcare, are generally preferred for their efficiency and sustainability [41]. More recent emerging evidence suggests that integrated approaches are the favoured direction and might be particularly beneficial for NTD management [41]_._ Notably, the Liberia study in this review demonstrated that integration itself was a key driver of cost-effectiveness: integrated case management of NTDs resulted in diagnosing and treating far more patients at a substantially lower cost per patient compared to fragmented, vertical programs, with the cost of diagnosis five times lower and treatment ten times lower under the integrated model.

In the management of NTDs, there is a notable division between preventive measures and treatment strategies. Preventive chemotherapy, such as cMDA, is widely recognized for its cost-effectiveness, particularly in controlling diseases like lymphatic filariasis and onchocerciasis [42]_._ This approach is generally more cost-effective compared to treatment, which often involves more complex and resource-intensive intervention [43]_._ However, a balance between prevention and treatment is crucial for comprehensive NTD management. CHWs play a pivotal role in bridging this gap by facilitating both preventive and treatment efforts. Our findings show that CHWs are mostly involved in initial screening for NTDs (n = 26 scenarios) and in delivery of medication (n = 26 scenarios), and less so in administrative tasks (n = 4 scenarios), outreach and training (n = 5 scenarios), and ongoing management (n = 3). Stronger Community Health Systems can also release demands placed on higher-level facilities, which is likely to be associated with additional health benefits. While treatment may be more complicated due to the need for individualized care and diagnostic tools, CHWs can help streamline these processes by providing health promotion, supporting self-treatment, and facilitating referrals. Their involvement in these holistic approaches could enhance the efficiency and reach of both preventive and treatment strategies, ultimately contributing to more effective NTD management - however more economic evidence is required to assess these different approaches.

### Strengths and Limitations

To our knowledge this is the only review to specifically document costs, cost-effectiveness and affordability of CHWs working to address NTDs in LMICs. As such, this research fills an important global knowledge gap, though the evidence base on these topics remains limited and prevents us from drawing firm conclusions about the cost-effectiveness or affordability of CHW-led programs focusing on NTDs. We also did not assess the quality of the included studies; however, this is in keeping with accepted scoping review methodology [44]_._

### Directions for future research

There are several important points that can be addressed in future research.

From a methodological perspective, studies should incorporate broader outcome measures (such as cost per DALY averted), different geographies, and explicitly state the analytic perspective and time horizon being used. For example, in the studies evaluated in this review, just over half (n = 17) adopted a period greater than one year. While this raises concerns about the adequacy of capturing long-term costs and benefits, particularly for interventions such as cMDA that may generate effects over extended periods, the shorter time horizon may have been appropriate given the aim of the research. Similarly, since NTDs often impose significant financial burdens on households, the choice of study perspective is important. While the majority of existing economic evaluation research on CHWs and NTDs adopts a health system perspective - valuable for informing government planning - this approach fails to capture the economic impact on households. Collecting and presenting evidence on the wider benefits of CHWs for NTDs would be valuable to advocate for more investments in CHW programs. Furthermore, the use of community-level care is likely to have downstream consequences, for instance through use of early diagnosis reducing future need for more complex and expensive healthcare, that don’t appear to be captured in the CEAs but warrant further investigation. Future research should study these possible effects further.

Finally, following the CHEERS checklist, when relevant, may help improve reporting quality and support better decision-making. We also recommend that future research prioritizes budget impact analyses or other approaches specifically designed to evaluate affordability, rather than concentrating exclusively on costs and cost-effectiveness.

## Conclusion

The economic evidence base on CHW involvement in NTD control has expanded since the Vaughan et al., (2015) review, but still remains highly limited with only 10 studies and no clear findings with regards to cost-effectiveness or affordability of CHWs in NTD programs in LMICs. A single study suggests that integrated interventions may be more cost-effective than vertical ones.

To effectively inform the vital role of CHWs in both prevention- and treatment-focused NTD programs, there is an urgent need for robust cost-effectiveness analyses and explicit affordability assessments. Without such evidence, it is challenging to make informed policy and funding decisions to optimize CHW contributions in NTD control.

## Supporting information

S1 Table Search strategies. (DOCX)

S2 Table PICO framework. (DOCX)

S1 Checklist PRISMA ScR checklist. (DOCX)

S2 Checklist Reflexivity statement. (DOCX)
