# Supplementary material for: Economic evaluations of community health worker programs focussed on neglected tropical diseases in low- and middle-income countries (2015–2024): A scoping literature review
Source: PLOS Glob Public Health. 2025 Dec 5;5(12):e0005551. doi: 10.1371/journal.pgph.0005551 (PMC12680331; doi:10.1371/journal.pgph.0005551)
Supplement: S2 Table — (DOCX) [file pgph.0005551.s002.docx]

# PICO framework

**Eligibility criteria - PICO framework**

| **Inclusion Criteria** | **Explanation** |
| --- | --- |
| **Population characteristics** | In this study we defined CHWs as healthcare workers who met the following three criteria:  (a) are primarily based in the community providing primary healthcare services  (b) part of the health system performing tasks related to health-care delivery, and/or health education, promotion, or care coordination  (c) have received organized training and/or certification, but do not have a tertiary-level degree such as a nursing or midwifery degree. |
| **Intervention** | Included studies focused on economic evaluations of CHW programmes in LMICs. For the purpose of this review we defined ‘economic evaluations’ as either:  **(a) Full Economic Evaluations** - this included (i) Cost-Effectiveness Analysis (CEA); (ii) Cost-Utility Analysis (CUA); (iii) Cost-Benefit Analysis (CBA); (iv) Cost-Minimization Analysis (CMA); (v) Cost-Consequence Analysis (CCA); (vi) Social Return on Investment (SROI); Multi-criteria decision analysis (MCDA); (vii) Budget Impact Analysis (BIA) and (viii) Programme Budgeting and Marginal Analysis (PBMA).  **(b) Partial Economic Evaluations** - this included studies reporting outcome description, cost description, cost outcome description effectiveness evaluation or cost analyses |
| **Comparator or Control treatment** | A specific comparator group was not necessary for inclusion; however, we did include studies which explored different modalities of CHW programming as comparison groups (for example volunteer programmes vs. professional programmes), in addition to those which had a “no intervention” comparator group. |
| **Setting, country, or jurisdiction** | This review focused on economic evaluations of CHW programmes in LMICs. For the purpose of this review we used the World Bank 2022 classification of economies to define countries as either low, lower-middle or upper-middle income. |
| **Outcomes** | We used the review as a way to explore the types and range of outcome measures used across the various forms of economic analysis.  In broad terms we were interested in social, environmental, economic and health outcomes valued in numerical or financial terms. In full economic evaluations, these were weighed against resource requirements to assess costs per unit improvements and incremental cost/cost-effectiveness.  The outcomes measures from:   - CEA includes: relative resource use, health outcome measures, incremental costs, and incremental cost-effectiveness ratio (ICER). - CUA includes: resource use; costs and ICER with costs expressed in monetary units and effects in quality-adjusted life-years (QALYs) or disability-adjusted life-years (DALYs). - CBA includes: relative resource use, costs and incremental net benefit ratio or net present value in relation to financial benefits, productivity gains and intangible benefits (e.g. individuals expressed willingness to pay). These were typically expressed in monetary values. - CMA includes: resource use and cost. - CCA includes : multiple outcomes indicated in the relevant studies will be considered including the cost and effects for different options taken. - SROI includes: social, economic, and environmental factors. - MCDA includes: clinical effectiveness, health-benefits, and cost-effectiveness which may be presented as individual or aggregate scores. - BIA includes: cost components within the budget - PBMA includes: economic analysis, multi-stakeholder inputs, values, needs and perspectives. - Cost analysis includes: resource use and cost. |

### 
